# 0726. The effects of carnitine on renal tissue damage and metabolic alteration among the rat intraabdominal sepsis model

**DOI:** 10.1186/2197-425X-2-S1-P48

**Published:** 2014-09-26

**Authors:** S Cetin, S Deniz, A Sezer, H Sen, S Ozkan

**Affiliations:** Department of Anesthesiology and Reanimation, Gulhane Military Medical Academy, Haydarpasa Training Hospital, Istanbul, Turkey

## Introduction

Sepsis is a systematic inflammatory reaction that causes renal damage frequently. Carnitine is considered as an essential mediator of metabolic pathway during sepsis and critical conditions. It has an important role in facilitating medium- and long-chain fatty acid transport from the cytosol into mitochondria for β-oxidation and energy generation [1].

## Objectives

We investigated the effects of carnitine usage on renal tissue damage and metabolic changes on rats with sepsis model. We hypothesized that carnitine decreases systemic inflammation and renal tissue damage.

## Methods

21 male rats randomly divided into 3 groups: sham operated (SO; n=7), sepsis (S; n=7) and sepsis + carnitine (S+C; n=7). In SO group only laparatomy was performed. In S and S+C groups caecal ligation and puncture was performed. At the postoperative 6^th^, 30^th^ and 54^th^ hours 1ml saline was administered intraperitoneally to both PO and S groups. Similarly 100mg/kg carnitine was administered intraperitoneally to S+C group at the postoperative 6^th^, 30^th^ and 54^th^ hours. After gathering blood and renal tissue samples, all rats were sacrificed at 60 hours postoperatively. Tissue malondialdehyde (MDA) levels were measured for oxidative stress and inflammation. Serum cytokines (TNF-α, IL-1, IL-6 and IL-10), neutrophil gelatinase-associated lipocalin (NGAL) and anti-caspase 3 were measured for inflammation markers, renal tissue damage and renal apoptosis, respectively.

## Results

In histopathological examination, S+C group has achieved better results in mononuclear cell infiltration score than the S group. Also, the increase in TNF-α, IL-1, IL-6, MDA, NGAL and anti-caspase 3 levels in S+C group were found to be lower than those of S group.

## Conclusion

Using carnitine in sepsis decreases the serum cytokine levels and renal damage and apoptosis via supressing the oxidative stress and inflammation both in blood and tissue.
